# DNA barcoding reveals the mislabeling of fish in a popular tourist destination in Brazil

**DOI:** 10.7717/peerj.4006

**Published:** 2017-11-29

**Authors:** Clisten Fátima Staffen, Mari Dalva Staffen, Mariana Londero Becker, Sara Emelie Löfgren, Yara Costa Netto Muniz, Renato Hajenius Aché de Freitas, Andrea Rita Marrero

**Affiliations:** 1 Biologia Celular, Embriologia e Genética (LAPOGE), Universidade Federal de Santa Catarina, Florianópolis, Santa Catarina, Brazil; 2 Ecologia e Zoologia (LABITEL), Universidade Federal de Santa Catarina, Florianópolis, Santa Catarina, Brazil

**Keywords:** Barcode, mtDNA, Japanese Cuisine, Brazil, Florianópolis, Fraud

## Abstract

The consumption of raw fish has increased considerably in the West, since it is said to be potentially healthier than processed fish (for containing omega 3 and 6, essential amino acids and vitamins). However this potential benefit, as well as the taste, value and even the risk of extinction are not the same for all species of fish, constituting grounds for fraud. Using the principles of the DNA barcode we revealed mislabelling of fish in Japanese restaurants and fishmarkets in Florianópolis, a popular tourist capital in Brazil. We sequenced the COI gene of 65 samples from fisheries and 80 from restaurants and diagnosed 30% of mislabeled samples in fisheries and 26% in restaurants. We discussed that frauds may have occurred for different reasons: to circumvent surveillance on threatened species; to sell fish with sizes smaller than allowed or abundant species as being a much rarer species (law of supply); to induce product consumption using species with better taste. It should be noted that some substitutions are derived from incorrect identification and are not a fraud *per se*; they are due to confusion of popular names or misunderstanding by the sellers. Therefore, we suggest the implementation of a systematic regulatory program conducted by governmental agencies to reduce mislabelling in order to avoid further damage to the community (in health and financial issues) and fish stocks.

## Introduction

Food fraud is an intentional adulteration to mask product conditions, or hide requirements that it does not meet, such as nutritional characteristics and price (Spink & Moyer, 2011). Since fish is a quickly decaying food, the main strategy to extend shelf life is to process the meat. The most common way to do so is filleting. The fillet is produced by cutting or slicing the flesh from the bone lengthwise, parallel to the backbone. This way many morphological structures are removed, making it difficult to recognize the species being cut, allowing accidental or intentional substitutions (Cawthorn, Steinman & Witthuhn 2012; Galimberti et al. 2013; Galal-Khallaf et al. 2014).

Accidental substitutions usually happen when species have similar morphological characteristics, the same vernacular name, or different names for the same species (Buck, 2007; Ardura et al., 2010; Barbuto et al., 2010; Cawthorn, Steinman & Witthuhn, 2012). On the other hand, intentional substitutions occur for the purpose of increasing profits by replacing species of high commercial value with species of low value or little market acceptance, as well as for the marketing of vulnerable or overexploited species (Logan et al. 2008; Cawthorn, Steinman & Witthuhn 2012; Huxley-Jones et al. 2012; Maralit et al. 2013; Cutarelli et al. 2014). There is strong evidence that intentional mislabeling of cheaper fish products is a more frequent phenomenon mainly within processed fish (Carvalho et al., 2015).

Such substitutions lead to problems associated with food security where substituted species pose a potential risk to human health (Handy et al. 2011; Galimberti et al. 2013), both the end consumer and the fishermen who were not intentionally involved in the fraud suffer economic losses (Ardura et al., 2010; Galimberti et al., 2013) and ecological implications that affect the conservation status of endangered and vulnerable species, leading to declining fish populations (Logan et al., 2008; Barbuto et al., 2010; Ardura, Planes & Garcia-Vazquez, 2013). Therefore, the aim here was to evaluate the authenticity of the identification of the fish sold in popular fish markets and Japanese restaurants in Florianópolis (Santa Catarina) through an efficient molecular tool such as DNA Barcode.

Hebert et al. (2003) proposed the DNA barcode as a sensitive and effective method of species identification. The comparative use of a 650 bp region of the mitochondrial Cytochrome Oxidase subunit I (COI) gene allows us to recognize it as being variable between species but very conserved in the species level. The COI DNA sequence can be obtained from a small biological sample and the method can be used in all living stages (this is particularly important in species that undergoes through metamorphosis). Although it cannot be used as a universal barcode gene as it is not well conserved in some organisms such as plants and protists, the DNA barcode stands as a valuable tool to identify not only phyla and order but also species as it is a small DNA region, easy to sequence, obtainable by a small biological sample, which offers researchers the possibility of identifying species through sequencing even though there is no preserved morphological feature (Wilson-Wilde et al., 2010).

## Material and Methods

### Samples

A total of 145 fish samples were collected from 12 Japanese Food Restaurants (JFR) and 09 fisheries in Florianópolis (southern Brazil), from July to November, 2015. A piece of 1 cm^3^ was fragmented in triplicates and stored in 96% ethanol at −20 °C until DNA extraction. Data was recorded, including date, location, type of fish product and common name of the species offered. The names of the establishments have been omitted to ensure confidentiality.

To minimize the chance of cross-contamination during collection, the fragments of fish chosen for sampling were cut and the inner part was sampled. The muscle of the fishwas collected with tweezers and scissors, sterilized with alcohol and disposable paper by a person wearing disposable gloves. A new set was used for each new sample collected, in order to avoid contamination.

This study is a continuation of a molecular surveillance program implemented by the Municipality of Florianópolis, previously described by Carvalho et al. (2015). The samples were taken in three sets, one of which was accompanied by officials from the PROCON—The Consumer Protection Program—a governmental regulatory agency. The other two sets were taken without prior notice to the establishment, with the sampler acting as a regular consumer.

### DNA extraction and sequencing

Genomic DNA was extracted from muscle tissue following the salting out protocol of Lahiri & Nurnberger (1991) with minor modification to reduce the final volume. We amplified the mitochondrial gene Cytochrome c oxidase subunit I (COI) using the primers L5698—Asn Forward 5′ AGG CCT CGA TCC TAC AAA GKT TTA GTT AAC 3′ (Miya & Nishida, 2000) and H7271—COI Reverse 5′ GTG GTG GGC TCA TAC AAT AAA 3′  (Melo et al., 2011). PCR mixtures consisted of 0.2 µL of Platinum Taq DNA Polymerase (5U/µL; Invitrogen, Carlsbad, CA, USA), 0.2 µL of each primer (10 pmol), 2.5 µL of 10× buffer, 1.5 µL of MgCl_2_ (50 mM), 1.0 µL of genomic DNA (20 ng/ µL) and purified water to complete the final reaction volume (25 µL). PCR conditions entailed 3 min at 94 °C, following 35 cycles of 30 s at 94 °C, 80 s at 56 °C, 160 s at 72 °C, finalized by 5 min at 72°C, after PCR was maintained at 4 °C. PCR products were visualized in 1% agarose gel for amplification check.

Positive reactions were purified with ExoSAP-IT (Exonuclease I: recombinant; SAP: *Pandalus borealis*—USB Corporation) and sequenced using the dideoxy-terminal method with Big Dye kit reagents (ABI PrismTM Dye Terminator Cycle Sequencing Reading Reaction; PE Applied Biosystems, Foster City, CA, USA) using an automatic capillary sequencer, model ABI3130 (Life Technologies, Carlsbad, CA, USA).

### Data analysis

Since there is significant confusion between popular names for the same species depending on the region in Brazil, we have adopted the names used in the Brazilian Normative Instruction No. 29 (Brasil & MAPA, 2015) while also comparing the corresponding ones to those vernacular names found on FishBase (http://www.fishbase.org).

The electropherograms were manually analyzed using the Chromas Lite 2.1.1 (http://www.technelysium.com.au) and sequences were checked and edited in BioEdit (Hall, 1999). The sequences were both double compared with GenBank (http://www.ncbi.nlm.nih.gov/genbank/) and the BOLD (www.boldsystems.org) databases employing Blastn Search Tool and BOLD identification tools, respectively. In all cases, BOLD was the criteria for the adopted species identification, only those with a similarity index greater than or equal to 98% in both databases were considered valid.

The sample was declared mislabeled if the species name, determined through molecular identification, did not match the commercially accepted name in this list. Additionally, we included the International Union for Conservation of Nature situation (IUCN Red List of Threatened Species status) obtained from http://www.iucnredlist.org and standardized from the Barcode Index Number sequence (BINs).

## Results and Discussion

The final sample was composed by 57 JFR samples and 88 fisheries samples. Tables 1 and 2 show the total of samples collected from each categories where no fraud was identified, which means that the product sold corresponded exactly with what was being stated (74% in JFR and 70% in fisheries).

**Table 1:** Summary of the 42 samples collected in restaurants in which sold species correspond to molecular identification species using DNA Barcoding. Only those with a match equal or greater to 98% were considered valid (both in BOLD and NCBI).

*n*	Sold as	Identified as	IUCN
29	Salmon	Salmon (*Salmo salar*)	LC
9	Tuna	Tuna (*Thunnus obesus*)	VU
1	“White fish”	Banded rudderfish (*Seriola zonata*)	LC
2	“White fish”	Dolphinfish (*Coryphaena hippurus*)	LC
1	“White fish”	Tilapia (*Oreochromis niloticus*)	NE

**Notes.**

Red List IUCN code NE Not evaluated DD Data deficient LC Least concern NT Near threatened VU Vulnerable EN Endangered

**Table 2:** Summary of the 62 samples collected in fisheries in which sold species correspond to molecular identification species using DNA Barcoding. Only those with a match equal or greater to 98% were considered valid (both in BOLD and NCBI).

*n*	Sold as	Identified as	IUCN
4	American harvestfish	American harvestfish (*Peprilus paru*)	LC
2	Blue Shark	Blue Shark (*Prionace glauca*)	NT
2	Bluefish	Bluefish (*Pomatomus saltatrix*)	VU
1	Corocoro grunt	Corocoro grunt (*Orthopristis ruber*)	LC
3	Croaker	Croaker (*Micropogonias furnieri*)	LC
1	Dolphinfish	Dolphinfish (*Coryphaena hippurus*)	LC
2	Escolar	Escolar (*Lepidocybium flavobrunneum*)	LC
5	Flounder	Flounder (*Paralichthys orbignyanus*)	NE
17	Salmon	Salmon (*Salmo salar*)	LC
1	Sardine	Sardine (*Sardinella aurita*)	LC
3	Shark (“Cação”)	Blue Shark (*Prionace glauca*)	LC
1	Shark (“Cação”)	Brazilian sharpnose shark (*Rhizoprionodon lalandii*)	DD
1	Shark (“Cação”)	Sandbar shark (*Carcharhinus plumbeus*)	VU
1	Shark (“Cação”)	Scalloped hammerhead (*Sphyrna lewini*)	EN
1	Swordfish	Swordfish (*Xiphias gladius*)	LC
1	Tilapia	Tilapia (*Oreochromis niloticus*)	NE
6	Tuna	Tuna (*Thunnus obesus*)	VU
10	Weakfish	Weakfish (*Cynoscion guatucupa*)	NE

**Notes.**

Red list IUCN code NE Not Evaluated DD Data deficient LC Least concern NT Near threatened VU Vulnerable EN Endangered

Although the quantity of samples is different between JFR and fisheries, applying an statistical test of adeherence (Chi square) we verify that the proportions are homogeneous even with different sample sizes, which allowed us to confirm that there is no significant difference in mislabeled samples proportion in fisheries and restaurants (*χ*^2^ = 0.229; *df* = 4; *α* = 0, 05).

When we turned our attention to the frauds (Tables 3 and 4) we noticed that, in the total analyzed sample, the general fraud rate was 28%. Considering separately JRF (26%) and fisheries (30%), we again have statistically homogeneous proportions. But what stands out that the pattern of fraud is a bit different since Brazilian JRF (targets of this research) traditionally offers only two well-defined species (Tuna and Salmon) and a third category (“white fish”) that is not related to any particular species. It is remarkable that the major motivation for these changes is apparently the cost, although some changes serve to cover other interests, such as the sale of Croaker as American Harvestfish. Both have similar economic value (around three dollars/kilo), but when Croaker are caught below average size, a common practice is filleting and selling it as a fish known to be smaller.

**Table 3:** Summary of the 15 samples collected in restaurants in which **mislabeling** was identified by DNA Barcoding. Only those with a match equal or greater to 98% were considered valid (both in BOLD and NCBI).

*n*	Sold as	Identified as	IUCN
1	Conger	Croaker (*Micropogonias furnieri*)	LC
1	Salmon	Tuna (*Thunnus alalunga*)	NT
2	Salmon	Weakfish (*Cynoscion guatucupa*)	NE
1	Salmon	Coho salmon (*Oncorhynchus kisutch*)	NE
1	Tuna	Banded rudderfish (*Seriola zonata*)	LC
2	Tuna	Escolar (*Lepidocybium flavobrunneum*)	LC
2	Tuna	Salmon (*Salmo salar*)	LC
1	Tuna	Yellowtail amberjack (*Seriola lalandii*)	LC
2	“White fish”	Tuna (*Thunnus obesus*)	VU
2	“White fish”	Salmon (*Salmo salar*)	LC

**Notes.**

Red list IUCN code NE Not Evaluated DD Data deficient LC Least concern NT Near threatened VU Vulnerable EN Endangered

**Table 4:** Summary of the 26 samples collected in fisheries in which **mislabeling** was identified by DNA Barcoding. Only those with a match equal or greater to 98% were considered valid (both in BOLD and NCBI).

*n*	Sold as	Identified as	IUCN
1	American harvestfish	Croaker (*Micropogonias furnieri*)	LC
1	Croaker	Blue Shark (*Prionace glauca*)	NT
1	Croaker	Weakfish (*Cynoscion guatucupa*)	NE
1	Escolar	Oilfish (*Ruvettus pretiosus*)	LC
1	Flounder	Bigtooth corvina (*Isopisthus parvipinnis*)	LC
2	Flounder	Croaker (*Micropogonias furnieri*)	LC
2	Flounder	Patagonian flounder (*Paralichthys patagonicus*)	NE
2	Flounder	Weakfish (*Cynoscion guatucupa*)	NE
1	Grouper	Croaker (*Micropogonias furnieri*)	LC
1	Grouper	Weakfish (*Cynoscion guatucupa*)	NE
1	Ling	Croaker (*Micropogonias furnieri*)	LC
1	Pangas catfish	Croaker (*Micropogonias furnieri*)	LC
1	Salmon	Banded rudderfish (*Seriola zonata*)	LC
1	Salmon	Blue Shark (*Prionace glauca*)	NT
1	Salmon	Croaker (*Micropogonias furnieri*)	LC
3	Sand tiger shark	Blue Shark (*Prionace glauca*)	VU/NT
1	Shark (“Cação”)	Weakfish (*Cynoscion guatucupa*)	NE
2	Swordfish	Largehead hairtail (*Trichiurus lepturus*)	LC
2	Weakfish	Bigtooth corvina (*Isopisthus parvipinnis*)	LC

**Notes.**

Red list IUCN code NE Not Evaluated DD Data deficient LC Least concern NT Near threatened VU Vulnerable EN Endangered

As previously mentioned, Brazilian JFR base their menu on three options: Salmon (popularly identified by the rosaceous hue), Tuna (dark meat) and “white fish”, which may vary in species. So, the color (and not the texture and flavor) seem to be the most frequent criteria, making fraudulent exchanges easier. In some establishments the popular name of the fish being sold as “white fish” were given. In these restaurants Dolphinfish, Weakfish or Tilapia were reported. Identification by barcode revealed that two of them were Dolphinfish, one case of Banded rudderfish and another of Tilapia. Since the expression “white fish” is broad, it is not appropriate to determine whether there was some kind of fraud because the color of the meat of these identified species correspond to this category (Table 1). However, two cases aroused attention (Table 3) where samples of “white fish” were identified as Salmon (twice) and Tuna (twice).

It is known that Salmon coloration is influenced by diet. The distribution of its color through the meat is not uniform, presenting a lighter shade in the muscles close to the horizontal septum, especially when there are fat infiltrations around the miosepta (Brasil & MAPA, 2016). This substitution is suggested to be motivated not by price differences but by taking advantage of all parts of the carcass, even if it is necessary to sell it as if it was some cheaper species. Strictly speaking, it is better to sell Tuna or Salmon as if it was a cheaper fish, like Yellow Amberjack, than to offer a product (part of the fish) that, because of its coloration, would not be recognized by the buyer as Salmon, and therefore be rejected.

Of all the collected samples, the most frequent is also the one of greater commercial value—Tuna, which is not a species, but a set of several of the same genus (this study identified two species: *Thunnus obesus* and *Thunnus alalunga*). A total of six Tuna frauds were identified in restaurants (and none in fisheries) and exchanges took place with four species: banded rudderfish (*Seriola zonata*), Salmon (*Salmo salar*), Yellowtail Amberjack (*Seriola lalandii*) and Escolar (*Lepidocybium flavobrunneum*).

Some replacements can not be regarded as fraud, but as a result of confusion between vernacular names. As an example it is worth mentioning the relationship between *Trichiurus lepturus* and *Xiphias gladius*, two very different kind of fish, not similar in shape, texture and even flavor, but both called “swordfish” in different regions of Brazil. The same is true for the fish known as Escolar, which may be the popular name for *Lepidocybium flavobrunneum* or *Ruvettus pretiosus* depending on the region of Brazil. These two species cause a gastrointestinal disease and are banned in some countries, like Japan, Italy and Republic of Korea, while other countries like Canada, Denmark and Sweden require health advisories and the European Union requires fishes to be appropriately labeled to provide information to consumers on adverse gastrointestinal effects (Dalama, Vieites & Espiñeira, 2015; Commission Regulation EC, 2005; Giusti et al., 2016). This draws attention to the importance of regulating the relationship between common names and their scientific names, especially for species of commercial interest.

The Atlantic salmon (*Salmo salar*) is a fish of high commercial value and an object of a very emphatic advertising campaign to increase its consumption. Among the 53 samples termed as Salmon, seven of them were identified as fraud (Tables 2 and 4). Such frauds are possible only by adding dye during the feeding process of farmed fish. In addition to the fraud related to market value, a more dangerous fraud is also present in these cases: it is known that cold-water fish like Tuna and Salmon are the best sources of polyunsaturated omega 3 fatty acids (Behs, 2011) which means that the consumer is not only deceived but their right to access quality food is also denied.

There is no information of direct damages to consumers’ health resulting from the frauds identified in this study, but there are a several reports of substitutions that caused damage to human health. An example published in 2002, where at least 63 people consumed herbal tea inadvertently mixed with neurotoxic Japanese star anise (*Illicium anisatum*) (Vermaak, Viljoen & Lindstron, 2013). Likewise, conditions as Hypercarotenemia (OMIM # 115300), an autosomal dominant disease, in which the main treatment consists of restrictive diet (Gangakhedkar, Somerville & Jelleyman, 2015) can cause several health damage to the individual who inadvertently consumes dyes used in feed farmed fish.

One sample was molecularly identified as Coho Salmon (*Oncorhynchus kisutch*), a fish of the Salmonidae family with meat color very similar to *Salmo salar*, but myoseptum not so well defined. This substitution was considered as a result of intentional fraud even though it may be only a misidentification.

In all substitutions, the most used species was the Croaker (*Micropogonias furnieri*) which was involved in nine types of fraud (22%). Since Croaker is a very common fish in the coastal area of southern Brazil, it takes significant part on the list of traded fishery species. Fillets coloration varies from white to pinkish, with predominantly white muscles, but with red muscles distributed along the horizontal caudal fin and septum. This tone variation from reddish to brown gives Croaker multiple exchange possibilities, as can be seen in Tables 2 and 4 in which Croaker fillet are identified being sold as Ling, Salmon, Panga Catfish, Conger, Grouper, Flounder, American Harvestfish and Shark. Although it is a very common species, the high replacement rate raises questions about the exploitation of fish stocks, since Croaker itself has a great demand and even fraudulently increases demand on other species.

In the opposite direction than expected, we also identified two frauds where Croaker itself was replaced by Weakfish and by Blue Shark, probably as a result of occasional increased fishing of these two species, since there is little difference in the amount of price by kilo (around three dollars).

The two samples collected as Grouper are from different establishments and both were mislabelled (by Weakfish and Croaker). Known for being a fish much appreciated in cooking, several species of Grouper are classified in the IUCN Red List of Threatened Species varying from NE (Near Threatened) to CE (Critically Endangered) due to destruction of their habitats and overfishing (Rosa & Lima, 2008). Due to the low fish stocks and great appreciation, Grouper is replaced by species with greater abundance and similar white meat when filleted.

Santa Catarina is the leading Brazilian state in Flounder (*Paralichthys orbignyanus*) capture, being one of the main targets of the fleet and one of the largest catches (Sampaio & Bianchini, 2002). In the present study, among the 12 samples sold as Flounder, two were rigged by Croacker (*Micropogonias furnieri*) and two by Weaker (*Cynosciuon guatupuca*) certainly aiming higher profitability.

The name “Shark” in Brazil has a stigmatized meaning, directly associated to an animal that eats people and garbage in the sea, with a stinking meat (high concentration of ammonia). Therefore, few people ask to buy Shark meat. Instead, Brazilian people prefer the name “Cação” for the fished and processed sharks. Even though the species are the same, the former does not have the stigmatized name and is more widely accepted as food (Bornatowski, Braga & Vitule, 2013). What is puzzling is that more than half of the population believes they are different animals (Bornatowski et al., 2015). Moreover, in Brazil there is no control that requires the identification of Shark species by commercial establishments and it is usually sold only with the generic term of “Cação” as we have shown in the present study. What is even worse in this scenery is the sale of endangered species, as seen here by the presence of *Sphyrna lewini* and *Carcharhinus plumbeus* (that are regarded as *Critically Endangered* species in the Brazilian coast following IUCN criteria— ICMBIO (2014)) simply as “Cação”. We also verified another fact that commonly happens in mislabeling: the selling of one species for another for financial gain and with potential to sell species at risk of extinction (Buck, 2007; Filonzi et al., 2010; Muñoz-Colmenero et al., 2016).

The fact that the fish sellers label the “Cação” sold as sandtiger shark (*Carcharias taurus*) is based on the fact that, historically, this species was often caught by spearfishing in the surrounding area of Santa Catarina State (Souza, 1994) and their meat was sold in the fish market. However, there is already clear evidence that the population of this species is in serious decline in Brazil nowadays, also regarded as “Critically Endangered” (ICMBIO, 2014). Another interesting fact in our study is the substitution of bone fish for shark and vice versa which is probably based on the law of supply and demand and possible financial gain. This was detected in a few cases in the literature, in which protected shark species were also sold as bony fish of high commercial value (Filonzi et al., 2010).

It is noteworthy that the fillet solded as Panga Catfish was identified as *Micropogonias furnieri* in both databases. To rule out the possibility of sampling error, a second DNA extraction and sequencing was arranged and the swap was characterized as an exceptional case of species of higher commercial value being sold for a lower price. While the kilo of Corvina costs more than three dollars, the kilo of Panga is below one dollar. Neto (2013) reported a similar substitution (Tilapia being sold as Panga Catfish) and suggested that this swap is a marketing strategy to induce product consumption. Panga Catfish is an imported species from Asia and is considered not to taste as good as Tilapia or Croaker, besides being fattier. Eventual substitutions with more palatable fish may mislead the consumer to consider it not so well cooked or seasoned instead of being a fraud.

These type of studies work with a direct application of knowledge, creating benefits for environmental issues by identifying environmental crimes and restraining them, like the restriction of species in illegal times or areas or even species at risk of extinction. Moreover, it allows the society to be safer concerning health issues related to seafood consumption.

Named as “Gato por Lebre”^1^ 1“cat by hare” is the Portuguese version of *“a pig in a poke”.*our project brings forth frauds and risks that consumers face, allowing the community to become well informed and enabling them to make better choices, in addition to directly reducing mislabeling levels in the long term.

## Conclusion

The study of mislabeling incidents in seafood markets brings more guarantees to consumers and also increases competitiveness among fishermen, sellers and restauranteurs who act within the norms. We believe that the implementation of a systematic regulatory program conducted by governmental agencies has a true impact in reducing market substitutions, bringing a direct benefit to society.

##  Supplemental Information

10.7717/peerj.4006/supp-1 Supplemental Information 1 Sequences generated from the fish samples (Florianópolis—Brazil)

## Additional Information and Declarations

### Data Availability

The following information was supplied regarding data availability: The dataset of sequences generated from the fish samples has been provided as a Supplemental File.
